# Supplementary material for: Incidence and Severity of COVID-19 in Relation to Anti-Receptor-Binding Domain IgG Antibody Level after COVID-19 Vaccination in Kidney Transplant Recipients
Source: Viruses. 2024 Jan 12;16(1):114. doi: 10.3390/v16010114 (PMC10820724; doi:10.3390/v16010114)
Supplement: Supplementary file 1 [file viruses-16-00114-s001.zip › viruses-2725368-supplementary.pdf]

# - Supplementary Materials -

Table of Contents

- **Supplementary Methods** – Questionnaire adherence to COVID-19 measures and restrictions
- **Table S1**. WHO COVID-19 Clinical Progression Scale - a measure of illness severity across a range from 1-10.
- **Table S2.** Characteristics of kidney transplant recipients included or excluded for analyses.
- **Table S3.** Characteristics of kidney transplant recipients with or without COVID-19 at follow-up.
- **Table S4.** Characteristics of kidney transplant recipients with severe COVID-19 or non-severe COVID-19 (i.e., a WHO CPS score of ≥4 or <4) during follow-up.
- **Table S5.** Mortality and hospitalisation rates of unvaccinated kidney transplant recipients in the Netherlands with COVID-19 when vaccination was not (yet) available versus vaccinated kidney transplant recipients.
- **Figure S1**. Percentage of infections per SARS-CoV-2 variant in the Netherlands (Data from National Institute for Public Health and the Environment (RIVM)) and number of COVID-19 cases in the study population according to date.

Supplementary Methods

Questionnaire adherence to COVID-19 measures and restrictions

Patients were asked to indicate to what extent they adhered to the following measures and restrictions after vaccination on a 5 point Likert scale (ranging from 1=never to 5=always): 1) Keeping distance of 1.5 meters from another 2) Wearing a face mask when mandatory or when social distancing is not possible 3) Washing hands 4) Avoiding supermarkets or other stores 5) Avoiding public transport 6) Avoiding crowded places 7) Limiting number of visitors or visits 8) Working from home 9) Avoiding travel abroad. The scores on the questions were summed and subsequently divided by the number of questions answered resulting in an average score with a minimum of 1 (never adhering to COVID-19 measures and restrictions) and a maximum of 5 (always adhering to COVID-19 measures and restrictions).

Table S1. WHO COVID-19 Clinical Progression Scale[1] - a measure of illness severity across a range from 1-10 (modified from Marshall et al. The Lancet Infectious Diseases, 2020).

**Score**	**Descriptor**	**WHO patient state**	**Classification for statistical analysis**
**1.**	Asymptomatic; viral RNA detected	Mild disease	Non-severe
**2.**	Symptomatic; independent		
**3.**	Symptomatic; assistance needed		
**4.**	Hospitalised; no oxygen therapy	Moderate disease	Severe
**5.**	Hospitalised; oxygen by mask or nasal progs		
**6.**	Hospitalised; oxygen by non-invasive ventilation or high flow	Severe disease	
**7.**	Intubation and mechanical ventilation, pO_2/FiO_2 ≥150 or SpO_2/FiO_2 ≥200		
**8.**	Mechanical ventilation, pO_2/FiO_2 <150 or SpO_2/FiO_2 <200 or vasopressors		
**9.**	Mechanical ventilation pO_2/FiO_2 <150 and vasopressors, dialysis or extracorporeal membrane oxygenation		
**10.**	Death	Death	

Table S2. Characteristics of kidney transplant recipients included or excluded for analyses.

	Included (n=2825)	*Missing, n (%)*	Excluded (n=943)	*Missing, n (%)*	P-value
Female, n (%)	1214 (42.1)	*0*	361 (38.3)	*0*	0.04
Caucasian, n (%)	2514 (92.2)	*157 (5.4)*	659 (87.2)	*187 (19.8)*	0.04
Age (years)	57.7 ± 12.9	*0*	55.6 ± 14.6	*3 (0.3)*	<0.001
BMI (kg/m^2)	26.1 ± 6.34	*134 (4.6)*	26.5 ± 4.72	*179 (19.0)*	0.11
Current smoking, n (%)	28 (1.0)	*108 (3.7)*	1 (0.1)	*172 (18.2)*	0.01
Current alcohol consumption, n (%)	1196 (43.1)	*112 (3.9)*	321 (41.6)	*171 (18.1)*	0.44
Number of comorbidities, n (%)		*300 (10.4)*		*176 (18.7)*	0.56
- 1	1329 (51.4)		378 (49.3)		
- 2	631 (24.4)		191 (24.9)		
- ≥3	337 (13.0)		114 (14.9)		
Comorbidities, n (%)					
- Hypertension	2323 (85.3)	*162 (5.6)*	638 (82.3)	*168 (17.8)*	0.04
- Diabetes Mellitus	549 (21.2)	*300 (10.4)*	215 (28.0)	*176 (18.7)*	<0.001
- History of coronary artery disease	307 (11.9)	*300 (10.4)*	97 (12.6)	*176 (18.7)*	0.57
- Heart failure	158 (6.1)	*300 (10.4)*	47 (6.1)	*176 (18.7)*	0.99
- Chronic lung disease	169 (6.5)	*300 (10.4)*	55 (7.2)	*176 (18.7)*	0.54
- History of malignancy	113 (4.4)	*300 (10.4)*	29 (3.8)	*176 (18.7)*	0.48
eGFR (ml/min/1.73m^2)	51.2 ± 18.7	*725 (25.1)*	51.9 ± 20.4	*217 (23.0)*	0.35
Primary renal diagnosis, n (%)		*670 (23.2)*		*187 (19.8)*	0.12
- Glomerulonephritis	455 (20.5)		152 (20.1)		
- Interstitial nephritis including pyelonephritis, drug induced nephropathy and urolithiasis	155 (7.0)		49 (6.5)		
- Cystic kidney diseases	400 (18.1)		114 (15.1)		
- Other congenital and hereditary kidney diseases	82 (3.7)		29 (3.8)		
- Renal vascular disease, excluding vasculitis	169 (7.6)		57 (7.5)		
- Diabetic Kidney Disease	129 (5.8)		63 (8.3)		
- Other multisystem diseases	115 (5.2)		37 (4.9)		
- Other	555 (25.1)		213 (28.2)		
- Unknown	155 (7.0)		48 (5.7)		
Transplant characteristics					
- First kidney transplant, n (%)	1843 (85.3)	*725 (25.1)*	613 (84.4)	*217 (23.0)*	0.56
- Time after last transplantation (years)	7 (3-13)	*725 (25.1)*	7 (3-12)	*219 (23.2)*	0.07
o ≤ 1 year, n (%)	237 (11.0)		73 (10.1)		0.50
- Last transplant					
o Living, n (%)	1368 (63.3)	*725 (25.1)*	476 (65.6)	*217 (23.0)*	0.28
o Pre-emptive, n (%)	824 (35.8)	*581 (20.1)*	267 (34.1)	*160 (17.0)*	0.40
Number of immunosuppressants, n (%)		*744 (25.8)*		*220 (23.3)*	0.13
- 1	82 (3.8)		34 (4.7)		
- 2	1071 (50.0)		332 (45.9)		

- ≥3	988 (46.1)		357 (49.4)		
Immunosuppressive treatment at baseline, n (%)		*744 (25.8)*		*220 (23.3)*	
- Steroids	1645 (76.8)		548 (75.8)		0.57
- Azathioprine	225 (10.5)		58 (8.0)		0.05
- Mycophenolate mofetil	1369 (63.9)		485 (67.1)		0.13
- Calcineurin inhibitor	1767 (82.5)		617 (85.3)		0.08
- mTor inhibitor	163 (7.6)		52 (7.2)		0.71
- Other	40 (1.9)		17 (2.4)		0.42
COVID-19 vaccination		*106 (3.7)*		*169 (17.9)*	<0.001
- mRNA-1273	2604 (93.7)		677 (87.5)		
- BNT162b2	117 (4.2)		71 (9.2)		
- ChAdOx1-S	56 (2.0)		25 (3.2)		
Adherence to COVID-19 restrictions*	4.25 (3.63-4.67)	*434 (15.0)*	4.0 (3.39-4.46)	*429 (45.5)*	<0.001
Social Economic Status**		*17 (0.6)*		*26 (2.8)*	0.65
- < -0.2	444 (15.5)		163 (17.8)		
- -0.2 – -0.1	257 (9.0)		79 (8.6)		
- -0.1 – 0	382 (13.3)		121 (13.2)		
- 0 – 0.1	517 (18.0)		158 (17.2)		
- 0.1 – 0.2	547 (19.1)		179 (19.5)		
- ≥0.2	721 (25.1)		217 (23.7)		

Variables are presented as mean ± SD in case of normal distribution, as median (IQ interval) in case of non-normal distribution or as absolute numbers and percentages in case of categorical data. P-values were calculated using independent *t* tests in case of normal distribution, Mann-Whitney U tests in case of non-normal distribution and Pearson's Chi squared tests in case of categorical data.

Abbreviations are: BMI, body mass index; eGFR, estimated glomerular filtration rate.

* Adherence to restrictions was determined by the average score on 9 questions on a 1-5 point Likert scale

**Social economic status was scored based on financial prosperity, educational level and recent employment history of households using data from publicly accessible data from Statistics Netherlands (CBS)[2]

Table S3. Characteristics of kidney transplant recipients with or without COVID-19 at follow-up.

	**COVID-19** (n=62)	**No COVID-19** (n=2823)	**P-value**
Female, n (%)	26 (41.9)	1188 (42.1)	0.98
Caucasian, n (%)	51 (87.9)	2463 (92.2)	0.23
Age (years)	56.4 ± 11.7	57.7 ± 12.8	0.38
BMI (kg/m^2)	26.3 ± 4.7	26.1 ± 6.4	0.86
Current smoking, n (%)	0	28 (1.0)	0.29
Current alcohol consumption, n (%)	26 (44.1)	1170 (43.1)	0.88
Number of comorbidities, n (%)			0.65
- 1	26 (45.6)	1303 (51.5)	
- 2	18 (31.6)	613 (24.2)	
- ≥3	7 (12.3)	330 (13.1)	
Comorbidities, n (%)			
- Hypertension	51 (86.4)	2272 (85.3)	0.80
- Diabetes Mellitus	19 (33.3)	530 (21.0)	0.02
- History of coronary artery disease	6 (10.5)	301 (11.9)	0.75
- Heart failure	3 (5.3)	155 (6.1)	0.79
- Chronic lung disease	4 (7.0)	165 (6.5)	0.88
- History of malignancy[1]	2 (3.5)	111 (4.4)	0.75
eGFR (ml/min/1.73m^2)	53.0 ± 19.5	51.2 ± 18.7	0.46
Primary renal diagnosis, n (%)			0.59
- Glomerulonephritis	15 (30.0)	440 (20.3)	
- Interstitial nephritis including pyelonephritis, drug induced nephropathy and urolithiasis	2 (4.0)	153 (7.1)	
- Cystic kidney diseases	9 (18.0)	391 (18.1)	
- Other congenital and hereditary kidney diseases	1 (2.0)	81 (3.7)	
- Renal vascular disease, excluding vasculitis	4 (8.0)	165 (7.6)	
- Diabetic Kidney Disease	4 (8.0)	125 (5.8)	
- Other multisystem diseases	1 (2.0)	114 (5.3)	
- Other	13 (26.0)	542 (25.0)	
- Unknown	1 (2.0)	154 (7.1)	
Transplant characteristics			
- First kidney transplant, n (%)	39 (79.6)	1804 (85.5)	0.25
- Time after last transplantation (years)	6 (4-14)	7 (3-13)	0.66
o ≤ 1 year, n (%)	3 (6.1)	234 (11.1)	0.27
- Last transplant			
o Living, n (%)	30 (61.2)	1338 (63.4)	0.76
o Pre-emptive, n (%)	16 (30.8)	808 (35.9)	0.45
Number of immunosuppressants, n (%)			0.26
- 1	4 (8.2)	78 (3.7)	
- 2	22 (44.9)	1049 (50.1)	
- ≥3	23 (46.9)	965 (46.1)	
Immunosuppressive treatment at baseline, n (%)			
- Steroids	32 (65.3)	1613 (77.1)	0.05
- Azathioprine	10 (20.4)	215 (10.3)	0.02

- Mycophenolate mofetil	31 (63.3)	1338 (64.0)	0.92
- Calcineurin inhibitor	42 (85.7)	1725 (82.5)	0.55
- mTor inhibitor	1 (2.0)	162 (7.7)	0.14
- Other	2 (4.1)	38 (1.8)	0.25
COVID-19 vaccination			0.90
- mRNA-1273	58 (96.7)	2546 (93.6)	
- BNT162b2	1 (1.7)	116 (4.3)	
- ChAdOx1-S	1 (1.7)	55 (2.0)	
Adherence to COVID-19 restrictions*	4.18 (3.43-4.64)	4.25 (3.67-4.67)	0.24
Social Economic Status**			0.19
- < -0.2	15 (24.2)	429 (15.3)	
- -0.2 – -0.1	3 (4.8)	254 (9.1)	
- -0.1 – 0	11 (17.7)	371 (13.2)	
- 0 – 0.1	12 (19.4)	505 (18.0)	
- 0.1 – 0.2	7 (11.3)	540 (19.2)	
- ≥0.2	14 (22.6)	707 (25.2)	
Anti-RBD IgG antibody level after vaccination (BAU/mL)	32.7 (2.93-350)	74.1 (10.9-612)	0.02

Variables are presented as mean ± SD in case of normal distribution, as median (IQ interval) in case of non-normal distribution or as absolute numbers and percentages in case of categorical data. P-values were calculated using independent *t* tests in case of normal distribution, Mann-Whitney U tests in case of non-normal distribution and Pearson's Chi squared tests in case of categorical data.
Abbreviations are: BMI, body mass index; eGFR, estimated glomerular filtration rate.

* Adherence to restrictions was determined by the average score on 9 questions on a 1-5 point Likert scale

**Social economic status was scored based on financial prosperity, educational level and recent employment history of households using data from publicly accessible data from Statistics Netherlands (CBS)[2]

Table S4. Characteristics of kidney transplant recipients with severe COVID-19 or non-severe COVID-19 (i.e., a WHO CPS score of ≥4 or <4) during follow-up.

	**Severe COVID-19** (n=15)	**Non-severe COVID-19** (n=47)	**P-value**
Female, n (%)	7 (46.7)	19 (40.4)	0.23
Caucasian, n (%)	13 (86.7)	38 (88.4)	0.86
Age (years)	59.8 ± 11.7	54.3 ± 11.4	0.06
BMI (kg/m^2)	25.8 ± 4.6	26.6 ± 5.3	0.31
Number of comorbidities, n (%)			0.19
- 1	3 (23,1)	23 (52.3)	
- 2	7 (53.8)	11 (25.0)	
- ≥3	2 (15.4)	5 (11.4)	
eGFR (ml/min/1.73m^2)	38.7 ± 17.3	58.3 ± 19.3	0.008
Transplant characteristics			
- First kidney transplant, n (%)	8 (88.9)	31 (77.5)	0.35
- Time after last transplantation (years)	4 (3-14)	7 (3-14)	0.64
o ≤ 1 year, n (%)	2 (22.2)	1 (2.5)	0.03
- Last transplant			
o Living, n (%)	2 (22.2)	28 (70.0)	0.008
o Pre-emptive, n (%)	1 (10.0)	15 (35.7)	0.11
Number of immunosuppressants, n (%)			0.94
- 1	1 (11.1)	3 (7.5)	
- 2	4 (44.4)	18 (45.0)	
- ≥3	4 (44.4)	19 (47.5)	
Immunosuppressive treatment at baseline, n (%)			
- Steroids	7 (77.8)	2 (22.2)	0.38
- Azathioprine	1 (11.1)	9 (22.5)	0.44
- Mycophenolate mofetil	7 (77.8)	24 (60.0)	0.32
- Calcineurin inhibitor	6 (66.7)	36 (90.0)	0.07
- mTor inhibitor	0	1 (2.5)	0.63
- Other	0	2 (5.0)	0.49
COVID-19 vaccination			0.71
- mRNA-1273	15 (100)	43 (95.6)	
- BNT162b2	0	1 (2.2)	
- ChAdOx1-S	0	1 (2.2)	
Adherence to COVID-19 restrictions*	4.38 (2.63-4.71)	4.14 (3.43-4.57)	0.93
Social Economic Status**			0.006
- < -0.2	1 (6.7)	14 (29.8)	
- -0.2 – -0.1	0	3 (6.4)	
- -0.1 – 0	5 (33.3)	6 (12.8)	
- 0 – 0.1	7 (46.7)	5 (10.6)	
- 0.1 – 0.2	1 (6.7)	6 (12.8)	
- ≥0.2	1 (6.7)	13 (27.7)	
Anti-RBD IgG antibody level (BAU/mL)	9.35 (1.18-23.6)	64.2 (5.41-425)	0.006

Variables are presented as mean ± SD or as median (IQ interval) dependent on data distribution or as absolute numbers and percentages in case of categorical data. P-values were calculated using independent *t* tests or Mann-Whitney U tests dependent on

data distribution and Pearson's Chi squared tests in case of categorical data. *Abbreviations are:* BMI, body mass index; eGFR, estimated glomerular filtration rate.

* Adherence to restrictions was determined by the average score on 9 questions on a 1-5 point Likert scale

**Social economic status was scored based on financial prosperity, educational level and recent employment history of households using data from publicly accessible data from Statistics Netherlands (CBS)[2]

Table S5. Mortality and hospitalisation rates of unvaccinated kidney transplant recipients in the Netherlands with COVID-19 when vaccination was not (yet) available versus vaccinated kidney transplant recipients.

	**Death[2]** N (%)	**Hospitalization** N (%)	**In hospital death** N (%)
Unvaccinated (n=247)[1]	48 (19.4)	139 (56.3)	43 (30.9)
Vaccinated (n=62)	4 (6.5)*	15 (24.2)**	4 (26.7)
• Seropositive (n=27)	1 (3.7)*	2 (7.4)**	1 (50.0)
• Seronegative (n=35)	3 (8.6)	13 (37.1)*	3 (23.1)

Variables are presented as absolute numbers and percentages; p-values were calculated using Pearson's Chi squared tests.

[1] Data from the second COVID-19 wave (July 2020 – April 2021) collected in ERACODA[3].

[2] Death was defined as all-cause mortality in kidney transplant recipients that were diagnosed with COVID-19 during follow-up; mortality rates in vaccinated patients may therefore be different compared to numbers mentioned in the main manuscript.

* P<0.05 compared to unvaccinated kidney transplant recipients

** P<0.001 compared to unvaccinated kidney transplant recipients

Figure S1. Percentage of infections per SARS-CoV-2 variant in the Netherlands (Data from National Institute for Public Health and the Environment (RIVM)) and number of COVID-19 cases in the study population according to date.

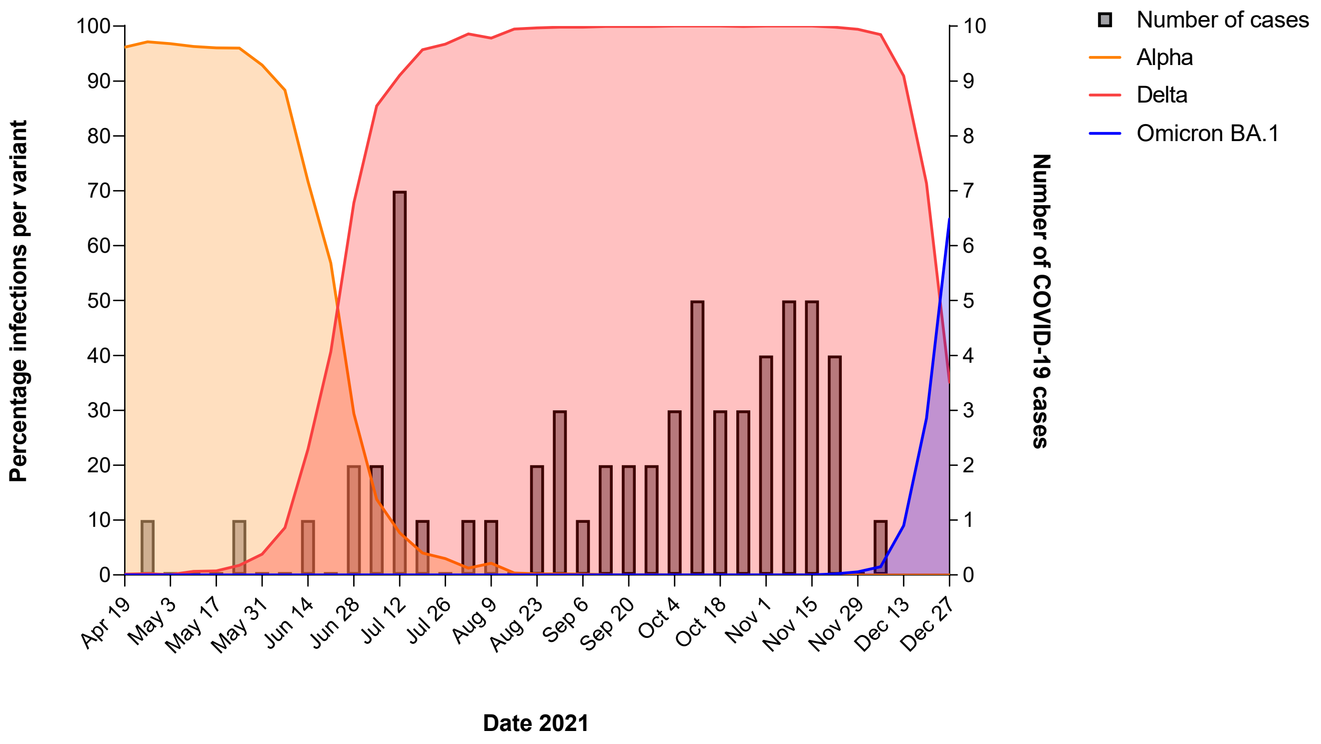

References

1. Marshall JC, Murthy S, Diaz J, et al. A minimal common outcome measure set for COVID-19 clinical research. *Lancet Infect Dis*. 2020;20(8):e192-e197. doi:10.1016/S1473-3099(20)30483-7

2. Centraal bureau voor statistiek. Sociaal-Economische Status; Scores per Viercijferige Postcode Op 1 Januari 2019. June 2022; https://www.cbs.nl/nl-nl/maatwerk/2022/26/sociaal-economische-status-per-postcode-2019

3. Noordzij M, Duivenvoorden R, Pena MJ, de Vries H, Kieneker LM. ERACODA: the European database collecting clinical information of patients on kidney replacement therapy with COVID-19. *Nephrol Dial Transplant*. 2020;35(12):2023-2025. doi:10.1093/ndt/gfaa179
